# Changes in Blood Pressure Levels and Antihypertensive Medication Use before and after Renal Transplantation among Patients in Nairobi, Kenya: A Comparative Cross-Sectional Study

**DOI:** 10.1155/2016/8450596

**Published:** 2016-12-07

**Authors:** Mary N. Kubo, Joshua K. Kayima, Anthony J. Were, Mohammed S. Ezzi, Seth O. McLigeyo, Elijah N. Ogola

**Affiliations:** ^1^Department of Clinical Medicine and Therapeutics, University of Nairobi (UoN), Nairobi, Kenya; ^2^Kenyatta National Hospital (KNH), Nairobi, Kenya

## Abstract

*Objectives. *To determine the changes in blood pressure levels and antihypertensive medication use in the postrenal transplantation period compared to pretransplantation one.* Methods*. A comparative cross-sectional study was carried out on renal transplant recipients at the Kenyatta National Hospital, a national referral hospital in Kenya. Sociodemographic details, blood pressure levels, and antihypertensive medication use before and after renal transplantation were noted. Changes in mean blood pressure levels and mean number of antihypertensive medications after renal transplantation were determined using paired *t*-test.* Results*. 85 subjects were evaluated. Mean age was 42.4 (SD ± 12.2) years, with a male : female ratio of 1.9 : 1. Compared to the pretransplant period, significantly lower mean systolic and diastolic blood pressure levels after transplantation were noted (mean SBP 144.5 mmHg versus 131.8 mmHg; mean DBP 103.6 mmHg versus 83.5 mmHg in the pre- and posttransplant periods, respectively, *p* < 0.001). Mean number of antihypertensive medications also reduced significantly after transplantation, with an average of 3.3 (±1.6) versus 2.1 (±0.9) in the pre- and posttransplant periods, respectively (*p* < 0.001).* Conclusion*. There is a significant reduction in blood pressure levels and number of antihypertensive medications used after renal transplantation. The positive impact of renal transplantation on blood pressure control should be confirmed using prospective cohort studies of patients with end stage renal disease who then undergo renal transplantation.

## 1. Background

Renal transplantation remains the therapeutic modality of choice for patients with end stage renal disease, with improved quality of life compared to dialysis. Blood pressure levels in patients with end stage renal disease are often elevated and difficult to control. The pathophysiology of hypertension in this population of patients is multifactorial, including volume overload, increased renin activity, enhanced sympathetic outflow, and increased blood viscosity due to erythropoietin replacement therapy [1]. Management of high blood pressure in this subset involves use of various antihypertensive agents, with diuretics playing a vital role in offloading the fluid overload status. Due to propensity to hyperkalemia as a result of deranged renal function, agents such as ACE inhibitors and ARBs are not routinely used as they cause further potassium retention.

The blood pressure achieved after transplantation is inversely related to postoperative glomerular filtration rate (GFR). As a result of improved GFR after transplant, there is significant improvement in blood pressure control, with fewer antihypertensive medications required. However the prevalence of postrenal transplant hypertension still remains high, with multiple mechanisms implicated [2]. In contrast to the general population, these mechanisms include both donor and recipient factors, including preexisting hypertension in the recipient, as well as donor age. In addition, transplant factors such as transplant dysfunction due to graft rejection and transplant renal artery stenosis also play a role. Cyclosporine commonly used as an immunosuppressant causes vasoconstriction leading to elevated blood pressure levels. Finally, glucocorticoids may also further exacerbate hypertension by causing sodium retention.

Unfortunately, despite manipulation of immunosuppressants used (by use of the lowest effective dose), most transplant recipients continue to require one or more antihypertensive agents to achieve adequate blood pressure control. Małyszko et al., in a study conducted on 150 renal allograft patients in Poland found that 60% of them required 3 or more antihypertensive agents, with only 40% demonstrating target blood pressure levels of less than 130/80 mmHg [3]. This is in contrast to the general, hypertensive population, whereby trials such as the ALLHAT Trial showed that an average of two drugs was required to achieve BP control (<140/90 mmHg) in two-thirds of patients [4].

There may be increased, reduced, or stable antihypertensive medication requirements after renal transplantation. A study carried out in India showed that up to 52% of posttransplant patients required 3 or more antihypertensive drugs, with most (62%) having increased antihypertensive requirements. Only 13% had reduced antihypertensive requirements after renal transplantation, while 25% had stable requirements [5].

In Norway, a study among nephrectomized renal transplant recipients showed that antihypertensive medication requirements reduced from a mean of 2.3 ± 0.5 drugs/day in the pretransplant period to 1.3 ± 0.9 drugs/day after renal transplantation [6].

The situation in Kenya regarding changes in blood pressure and antihypertensive medications in the posttransplant period compared to the pretransplant period remains unknown. This study sought to document these changes.

## 2. Methods

### 2.1. Study Design

It is comparative cross-sectional study.

### 2.2. Setting

It is conducted in Kenyatta National Hospital Renal Transplant Clinic. Kenyatta National Hospital is a tertiary referral hospital and runs a specialized renal transplant clinic.

### 2.3. Participants

The entire cohort of renal transplant recipients was hypertensive, more than 2 months after transplant, 18 years of age, and above and not on dialysis due to nonfunctional grafts and gave informed written consent.

### 2.4. Sample Size

All eligible patients attending the nephrology clinics and fulfilling the inclusion criteria were included.

### 2.5. Sampling Method

All eligible patients were consecutively sampled.

### 2.6. Objectives

- First, it is to compare blood pressure levels in the pre- and postrenal transplant periods.
- Second, it is to compare number and type of antihypertensive medications used in the pre- and postrenal transplant periods.

### 2.7. Definitions

*Hypertension* was defined as either the use of antihypertensive therapy* or* systolic blood pressure ≥140 mmHg and/or diastolic blood pressure ≥90 mmHg. Controlled blood pressure was defined as BP < 130/80 mmHg as per KDIGO guidelines [7].

*Antihypertensive medication use* was defined as the number of different* classes* of antihypertensive medications used for management of blood pressure in both the pre- and posttransplant periods.

### 2.8. Data Collection

Informed written consent was obtained prior to patient enrolment into the study. Participants' sociodemographic history and clinical history were then recorded in a predesigned questionnaire. Blood pressure measurement on the day of enrolment was done using standard procedure with duly calibrated machines [8]. In addition, blood pressure readings from the preceding two clinic visits were also recorded. An average of the three blood pressure readings was then calculated. Pretransplant blood pressure readings were abstracted from patient records. An average of three blood pressure readings from the last three clinic visits prior to renal transplantation was calculated from these records.

### 2.9. Statistical Analysis

For the baseline characteristics, continuous variables were summarized into means and standard deviations (SD) or medians and interquartile ranges (IQR) while categorical variables were presented as proportions.

Antihypertensive medication use was analyzed and presented as mean number of drugs used per patient. Changes in antihypertensive medication use after transplantation were determined by comparing the mean number of drugs before and after transplantation using paired *t*-test. Changes in blood pressure levels after transplantation were also determined by comparing the mean blood pressure before and after transplantation using paired *t*-test. All statistical tests were performed at 5% level of significance.

### 2.10. Ethical Issues

Ethical approval was given by the KNH/UoN Ethics and Research Committee, and all procedures were in accordance with the institutional ethical standards. Patients gave informed written consent. Confidentiality of patient records was maintained at all times.

## 3. Results

### 3.1. Baseline Characteristics of Study Participants

Out of 100 documented renal transplant recipients attending the nephrology clinics, 85 were found to be eligible and recruited into the study. Data for all 85 study participants was subsequently analysed. This is presented in Table 1.

The average age of the renal transplant recipients was 42.4 (±12.2) years, with the donors being younger at a mean age of 33.2 (±8.5) years. Most recipients were male (65.9%), with a male to female ratio of 1.9 : 1. The male predominance was also noted among the donors, 54.1% of whom were male.

### 3.2. Blood Pressure Levels and Control

In the pretransplant period, uncontrolled hypertension was noted in 87.1% of the patients (95% CI, 78.9–94.1), while in the posttransplant period 68.2% (95% CI, 57.6–77.6) had uncontrolled hypertension (Table 2).

Compared to the pretransplant period, significantly lower mean systolic and diastolic blood pressure levels were noted after transplantation (mean SBP 144.5 mmHg versus 131.8 mmHg; mean DBP 103.6 mmHg versus 83.5 mmHg in the pre- and posttransplant periods, respectively, *p* < 0.001) (Table 2).

**Figure 1:**
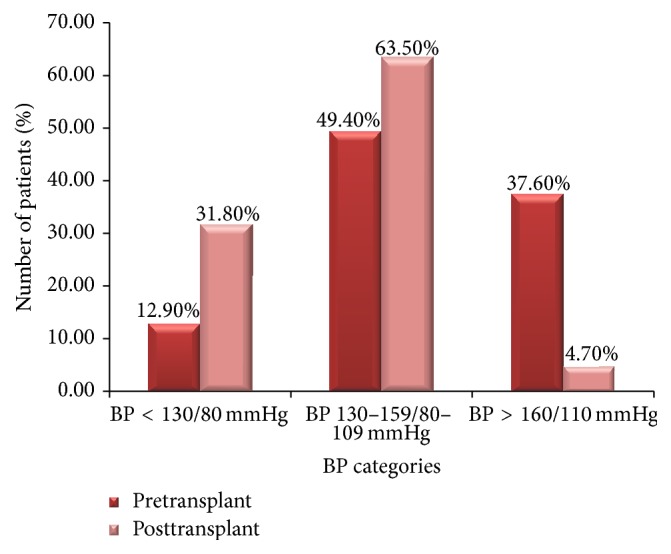
Blood pressure categories before and after transplant.

It was noted that in the pretransplant period more than a third of the patients (37.6%) had blood pressure levels greater than 160/110 mmHg. This was however reduced to 4.7% in the posttransplant period (Figure 1).

### 3.3. Antihypertensive Medications

**Figure 2:**
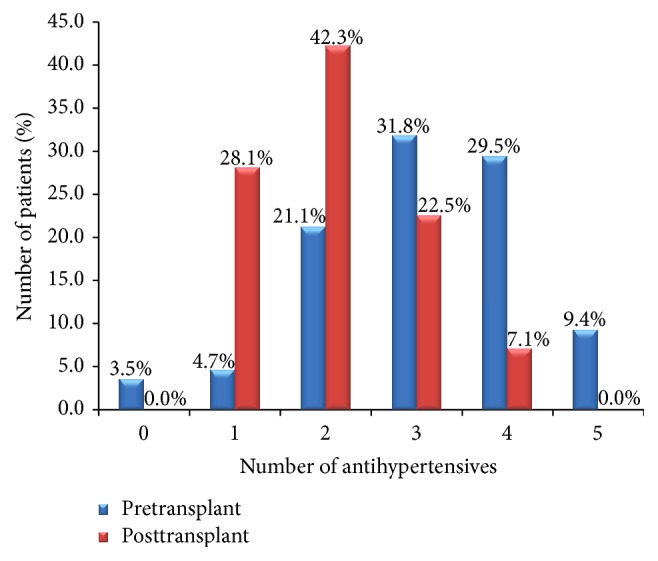
Number of antihypertensives used in the pre- and posttransplant periods.

In the pretransplant period most patients (70.7%) were on 3 or more classes of antihypertensive medications. In the posttransplant period the converse was noted, with most patients (70.4%) on 2 or less antihypertensive medications (Figure 2).

**Figure 3:**
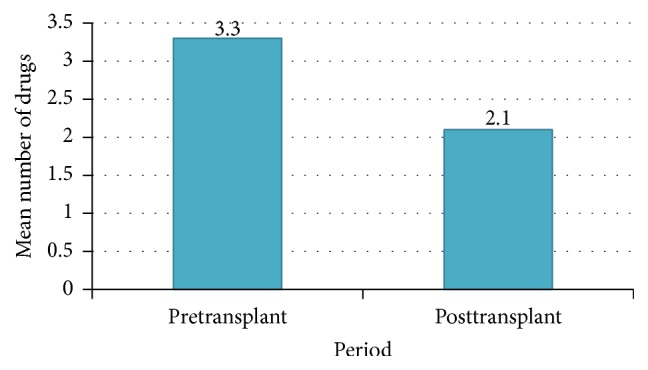
Mean number of antihypertensives used in the pre- and posttransplant periods.

There was a significant reduction in the mean number of antihypertensive agents used after transplantation, with an average of 3.3 (±1.6) drugs in the pretransplant period compared with 2.1 (±0.9) drugs in the posttransplant period (*p* < 0.001). This is shown in Figure 3.

### 3.4. Types of Antihypertensive Agents Used

**Figure 4:**
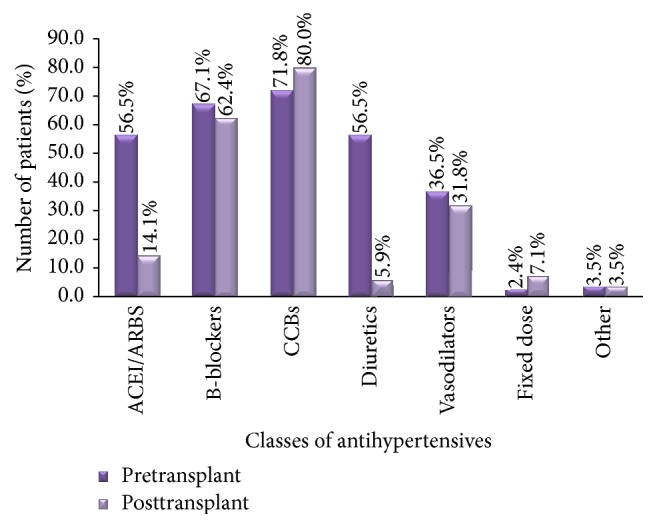
Types of antihypertensive drugs used before and after transplant.

With regard to the types of antihypertensive agents used, it was noted that there was a marked reduction in ACE inhibitor use from 56.5% in pretransplant to 14.1% in the posttransplant period, as well as diuretic use (56.5% to 5.9%) (Figure 4).

Use of calcium channel blockers increased slightly in the posttransplant period from 71.8% to 80%, as did use of fixed dose combinations (2.4% to 7.1%). Beta-blocker and vasodilator use remained almost similar before and after transplantation.

The most commonly used classes of antihypertensives were calcium channel blockers and beta-blockers.

## 4. Discussion

Blood pressure control in our renal transplant population still remains a challenge, with 68.2% of renal transplant recipients in our study having uncontrolled hypertension. In comparison, studies in the West showed much lower rates of uncontrolled hypertension, with figures of 50% in UK [9] and 58% in Jordan [10]. This difference could be attributed to the fact that their populations had access to free medications. In addition, their mean duration after transplantation was much longer than ours. Studies have demonstrated that blood pressure control tends to improve as time after transplantation increases [11]. This is because of stabilization of graft function as well as tapering off of immunosuppressant doses, especially cyclosporine and steroids that have been implicated in causation of hypertension.

As compared to the posttransplant period, there were higher rates of uncontrolled hypertension in the pretransplant period, with 87.1% of the patients not achieving target blood pressure goals. It is well established that higher blood pressure levels are seen with decreased or worsening renal function [12].

Reasons for better rates of blood pressure control in the posttransplant period include the fact that blood pressure after renal transplantation is inversely related to the glomerular filtration rate. Many patients thus note a reduction in blood pressure levels with the improvement in renal function achieved after transplantation, as well as the reduced fluid overload status. Indeed, mean systolic and diastolic pressure were noted to decrease significantly from 144.5 (±18.2) mmHg to 131.8 (±16.6) mmHg and 103.6 (±37.7) mmHg to 83.5 (±12.9) mmHg in the pre- and posttransplant periods, respectively.

In terms of antihypertensive medication use, it was noted that there was a significant reduction in mean number of antihypertensives used from 3.3 (±1.6) in the pretransplant period to 2.1 (±0.9) in the posttransplant period. This was also noted in a study carried out by Midtvedt et al. in Norway, where antihypertensive medication use reduced from 2.3 (±0.3) to 1.3 (±0.9) among renal transplant recipients who also underwent bilateral nephrectomy [6]. Similar reduction in antihypertensive use was noted in both the patients who had nephrectomy and those who did not. Reduced antihypertensive medication use is attributed to the lower blood pressure levels seen with improved GFR after renal transplantation.

The most commonly used class of antihypertensive agents was calcium channel blockers in both the pre- and posttransplant period, followed by beta-blockers. Bulatova et al. in Jordan also had similar findings, with 58% of their patients on CCBs followed by beta-blockers at 33% [10]. Calcium channel blockers are preferred agents especially in the early posttransplant period because they have been shown to mitigate the vasoconstrictive effects of cyclosporine. A large randomized comparative study also showed that nifedipine was associated with improved kidney function at one year after transplant when compared to the ACE inhibitor, lisinopril [13].

There was a marked reduction in ACE inhibitor/angiotensin receptor blocker use from 56% in the pretransplant period to 14% in the posttransplant period. This reflects the fact that ACEi/ARBs are usually avoided in the immediate posttransplant period because they may cause a reduction in glomerular filtration rate that may mimic or mask rejection episodes. They may also precipitate renal failure in patients with transplant renal artery stenosis. Bulatova et al. also noted that only 19% of their renal transplant recipients were on ACE inhibitors [10]. In addition, we also observed a decline in diuretic use after transplantation (56.5% to 5.9%), probably as a result of the reduced fluid overload status achieved with improved renal function after transplantation.

One of the limitations of this study is that pretransplant blood pressure levels were abstracted retrospectively from file records, which may be affected by interobserver variability in measurement of blood pressure during clinic visits.

## 5. Conclusions

Uncontrolled hypertension remains highly prevalent at 68.2% in the postrenal transplant period, although there was an improvement in blood pressure control when compared to the pretransplant period.

It was also noted that there was a reduction in mean number of antihypertensive medications used in the posttransplant period compared to the pretransplant period.

Prospective cohort studies following up patients with end stage renal disease who then undergo renal transplantation will be best suited to confirm the positive impact of renal transplantation on antihypertensive medication requirements and blood pressure levels indicated in this comparative cross-sectional study.

## Figures and Tables

**Table 1:** Baseline characteristics.

Variables	Frequency (%)
*Age in years (recipient)*	
Mean (SD)	**42.4** (12.2)
Min-max	18–68
*Sex (recipient)*	
Male	56 (**65.9)**
Female	29 (34.1)
*Age in years (donor)*	
Mean (SD)	**33.2** (8.5)
Min-max	21–54
*Health insurance*	
Yes	85 (**100**)
*Serum creatinine (µmol/L)*	
Mean (SD)	118.2 (37.2)
Min-max	69–321
*Urine albumin : creatinine ratio (mg/g)*	
<30	40 (47.1)
30–300 (microalbuminuria)	36 (42.4)
>300 (macroalbuminuria)	9 (10.5)
*Mean BMI in kg/m* ^*2 *^ *(SD)*	24.8 (4.4)
*History of acute rejection*	
Yes	14 (6.4)
No	71 (83.6)

**Table 2:** Blood pressure levels and control.

Variable	Pretransplant	Posttransplant	*p* value
Blood pressure, mean (SD)			
Systolic	144.5 (18.2)	131.8 (16.6)	**<0.001**
Diastolic	103.6 (37.7)	83.5 (12.9)	**<0.001**
Hypertension control, *n* (%)			**0.006**
Uncontrolled	74 (87.1%)	58 (68.2)	
Controlled	11 (12.9%)	27 (31.8)	
